# Diabetes Increases the Vulnerability of the Cardiac Mitochondrial Network to Criticality

**DOI:** 10.3389/fphys.2020.00175

**Published:** 2020-03-10

**Authors:** Larissa Vetter, Sonia Cortassa, Brian O’Rourke, Antonis A. Armoundas, Djahida Bedja, Johann M. E. Jende, Martin Bendszus, Nazareno Paolocci, Steven J. Sollot, Miguel A. Aon, Felix T. Kurz

**Affiliations:** ^1^Laboratory of Cardiovascular Science, National Institute on Aging, National Institutes of Health, Baltimore, MD, United States; ^2^Department of Neuroradiology, Heidelberg University Hospital, Heidelberg, Germany; ^3^Division of Cardiology, Department of Medicine, Johns Hopkins University, Baltimore, MD, United States; ^4^Cardiovascular Research Center, Massachusetts General Hospital, Harvard Medical School, Charlestown, MA, United States; ^5^Institute for Medical Engineering and Science, Massachusetts Institute of Technology Cambridge, MA, United States; ^6^Department of Biomedical Sciences, University of Padua, Padua, Italy

**Keywords:** type 1 diabetes, cardiac myocyte, mitochondrial criticality, mitochondria, wavelet analysis

## Abstract

Mitochondrial criticality describes a state in which the mitochondrial cardiac network under intense oxidative stress becomes very sensitive to small perturbations, leading from local to cell-wide depolarization and synchronized oscillations that may escalate to the myocardial syncytium generating arrhythmias. Herein, we describe the occurrence of mitochondrial criticality in the chronic setting of a metabolic disorder, type 1 diabetes (T1DM), using a streptozotocin (STZ)-treated guinea pig (GP) animal model. Using wavelet analysis of mitochondrial networks from two-photon microscopy imaging of cardiac myocytes loaded with a fluorescent probe of the mitochondrial membrane potential, we show that cardiomyocytes from T1DM GPs are closer to criticality, making them more vulnerable to cell-wide mitochondrial oscillations as can be judged by the latency period to trigger oscillations after a laser flash perturbation, and their propensity to oscillate. Insulin treatment of T1DM GPs rescued cardiac myocytes to sham control levels of susceptibility, a protective condition that could also be attained with interventions leading to improvement of the cellular redox environment such as preincubation of diabetic cardiac myocytes with the lipid palmitate or a cell-permeable form of glutathione, in the presence of glucose.

## Introduction

Mitochondrial dysfunction can lead to energy depletion and electrical instability in the heart, increasing the vulnerability to arrhythmias (Akar et al., 2005; O’Rourke et al., 2005). Energy depletion can cause a rapid activation of ATP-sensitive K^+^ (K_ATP_) channels that leads to action potential duration (APD) shortening (O’Rourke et al., 1994; Aon et al., 2003; Zhou et al., 2009, 2014). *Mitochondrial criticality*, a seminal event upstream of K_ATP_ channel activation, leads to an abrupt decrease in energy supply (Aon et al., 2004a, 2006; O’Rourke et al., 2005). Oxidative stress drives the state of the mitochondrial network to *criticality*, a situation in which even small perturbations will trigger cell-wide depolarization in the form of a propagated mitochondrial membrane potential (ΔΨ_m_) depolarization wave followed by sustained, self-organized, low frequency (i.e., long period) and high amplitude oscillations of ΔΨ_m_ (Aon et al., 2003; Cortassa et al., 2004). The rapid ΔΨ_m_ depolarization converts mitochondria from generators into consumers of ATP, leading to a decrease of the cellular ATP/ADP ratio, with consecutive activation of K_ATP_ channels and shortening of the APD (O’Rourke et al., 1994; Aon et al., 2003, 2009; Zhou et al., 2009). More recently, it has been shown that mitochondrial ΔΨ_m_ oscillations are consistently observed in cardiomyocyte monolayers upon reperfusion after a period of ischemia. They provoke spontaneously developing reentrant arrhythmias during ΔΨ_m_ recovery (Solhjoo and O’Rourke, 2015). Since the instability provoked by mitochondrial criticality can escalate to the cardiomyocyte and whole heart levels, potentially provoking life-threatening arrhythmias, the importance of understanding the conditions leading to this phenomenon cannot be overstated.

Diabetes mellitus is a frequent metabolic disorder with several complications that are typically associated with macro- and microangiopathic alterations (Madonna et al., 2017; Jende et al., 2019a). Increased markers of microangiopathy have been implicated in peripheral neuropathy and cardiac myopathy, and linked to mitochondrial dysfunction (Amano, 2016; Jia et al., 2018; Smyrnias et al., 2019; Jende et al., 2020). Although an impressive body of knowledge exists about the role played by mitochondrial dysfunction in diabetes, the mechanisms underlying their impact on diabetic cardiomyopathy, a severe and potentially lethal complication that limits T1DM patients’ life expectancy and quality of life, are still not well understood (Aon and Foster, 2015). An altered pro/anti-oxidant balance and changes in lipid metabolism are mainly associated with the onset/progression and pathological consequences of diabetes (Aon et al., 2015; Galloway and Yoon, 2015; Hafstad et al., 2015; Schilling, 2015; Jende et al., 2019b). In type 1 and type 2 diabetes, mitochondrial function has to adapt to oxidative stress generated by increased levels of circulating glucose and lipids (Aon et al., 2014; Roul and Recchia, 2015; Sung et al., 2015; Schrauwen-Hinderling et al., 2016), the impairment of mitochondrial antioxidant capacity (Tocchetti et al., 2012; Della Rosa et al., 2013), and metabolic peaks of glucose and concomitant adrenergic hyperactivity (Tocchetti et al., 2012, 2015; Della Rosa et al., 2013; Mebazaa et al., 2013; Bhatt et al., 2015). Metabolic substrates influence the dynamic behavior of the mitochondrial cardiac network by altering its catabolic pathways and the ROS/redox balance (Kurz et al., 2015). In this regard, recent work showed that in intact heart under glucose excess, acute palmitate addition elicits metabolic remodeling leading to enhanced redox status, which is linked to improved contractility/relaxation in diabetic *db*/*db* mice, compared to their non-diabetic controls, when subjected to stress given by β-adrenergic stimulation with isoproterenol (Cortassa et al., 2018). Consequently, the concurrent effects of the abovementioned diabetic conditions increase the likelihood of mitochondrial criticality.

In the present work, we explore the occurrence of mitochondrial criticality in the chronic setting of a metabolic disorder, T1DM, using a previously described streptozotocin (STZ)-treated guinea pig animal model that harbors glucose levels similar to those found in human T1DM (Tocchetti et al., 2015). We hypothesized that the mitochondrial network in cardiac myocytes from T1DM GPs is closer to criticality, compared to non-diabetic controls, rendering them more prone to the triggering of synchronized whole-cell mitochondrial oscillations. Wavelet analysis of the dynamic behavior of mitochondrial networks has provided insights into mechanisms of coupling and synchronization under various conditions of substrate availability and stress (Kurz et al., 2010a, b, 2015). Using wavelet analysis as applied to mitochondrial networks, combined with two-photon laser scanning fluorescence microscopy, we found that, compared to Sham controls, cardiomyocytes from T1DM GPs are, indeed, closer to criticality, making them more susceptible to whole-cell oscillations. Insulin treatment of T1DM GPs rescued cardiac myocytes to Sham control levels of vulnerability, a protective condition that could also be attained by preincubation of diabetic cardiomyocytes with the lipid palmitate or the cell permeable glutathione ethyl ester (GSHee) in the presence of glucose.

## Materials and Methods

### Diabetic Guinea Pig (Cavia porcellus)

Diabetic GPs were generated by Hilltop Lab Animals, Inc. (Scottdale, PA, United States), as described elsewhere (Tocchetti et al., 2015). All procedures were approved by the Animal Care and Use Committee of Hilltop Lab Animals, Inc., and adhered to NIH public health service guidelines. Sham and STZ animals were utilized after 4 weeks of STZ administration, whereas the STZ+ Ins group was treated for another 2 weeks with insulin and then analyzed. Briefly, a single intraperitoneal injection of buffered streptozotocin was used to render male GPs (200–250 g) diabetic (*STZ group*, 80 μg/kg in citrate buffer pH 4.5). Age-matched littermate GPs were given an equivalent amount of vehicle (*Sham group*, citrate buffer pH 4.5). Four weeks after STZ injection, the GPs from the insulin-treated STZ group (*STZ* + *Ins*) received 1 Unit/day of Insulin glargine (Lantus, Aventis) subcutaneously for another 2 weeks. Diabetic GPs (STZ group) had 36% higher levels of glucose in blood (*P* < 0.001 with respect to controls (Sham group) and STZ + Ins, i.e., from ∼8 to ∼12 mM glucose) (Tocchetti et al., 2015). Insulin addition normalized glucose levels as revealed by fasting blood glucose levels (mg/dl + S.E.M.) Sham: 153 ± 3.2 (*n* = 26); STZ: 208 ± 6.5 (*n* = 24); STZ + Ins: 155 ± 5 (*n* = 17).

### Myocyte Isolation

For cardiomyocyte isolation, GPs were heparinized (500 IU) and euthanized with sodium pentobarbital (180 μg/kg intraperitoneal), following the requirements of the Institutional Animal Care/Use Committee at JHU, adherent to NIH guidelines (Tocchetti et al., 2015). The isolated heart was cannulated through the aorta allowing for retrograde perfusion and ventricular myocytes preparation as described (O’Rourke et al., 1994; Aon et al., 2003).

### Two-Photon Laser Scanning Fluorescence Microscopy of Isolated Cardiomyocytes

All experiments were carried out with ventricular myocytes freshly isolated from GP hearts. Cardiomyocytes from Sham, STZ or STZ + Ins-treated GPs were loaded with 100 nM tetramethylrhodamine ethyl ester (TMRM), a cationic fluorescent dye to observe the mitochondrial membrane potential (ΔΨ_m_), at 37°C in a thermostatically controlled flow chamber mounted on the stage of an upright epifluorescence microscope (BX61WI; Olympus, Waltham, MA, United States) (Aon et al., 2003, 2007). Myocytes were visualized with an objective 25x/1.05 W MP and images were taken every 1.644 s or 3.5 s with a multiphoton-excited fluorescence Fluoview FV1000 MPE (Olympus) and a Deep Sea ultrafast system scanning laser (Mai Tai, Waltham, MA, United States), with excitation at 740 nm and red emission of TMRM collected at 605 nm, using a 578–630 nm band-pass filter (Kurz et al., 2015).

### Evaluation of the Cardiomyocyte Propensity to Exhibit Mitochondrial Oscillations

Cardiomyocytes were perfused with Tyrode solution (pH 7.5) that contained 1 mM Ca^2+^ and 10 mM glucose, and their propensity to exhibit mitochondrial oscillations was investigated by a localized, single laser flash comprising a small region of a cardiac myocyte (20 × 20 pixels, 8.7 × 8.7 μm^2^, 81 μm^3^, ∼30–40 mitochondria) (Aon et al., 2003, 2004a). In addition to glucose, and when indicated (e.g., Table 1), cardiomyocytes isolated from control (Sham) or diabetic (STZ) guinea pigs were preincubated for at least 2 h with 4 mM glutathione ethyl ester (GSHee), a cell-permeable form of glutathione (GSH), whereas another group of cardiac myocytes from diabetic GPs was incubated with 0.4 mM palmitate (Palm), in both cases in addition to glucose.

**TABLE 1:** Susceptibility to laser flash-induced oscillations exhibited by ventricular myocytes from guinea pig hearts.

	Sham	Diabetic	Diabetic + Insulin
		(STZ)	(STZ+Ins)
Control	154/50 (32%) (*n* = 8)	200/118 (59%) (*n* = 8)	126/46 (36%) (*n* = 4)
+4 mM GSHee	65/20 (31%) (*n* = 4)	80/24 (30%) (*n* = 4)	–
+0.4 mM Palm	–	76/25 (33%) (*n* = 4)	–

**FIGURE 1:**
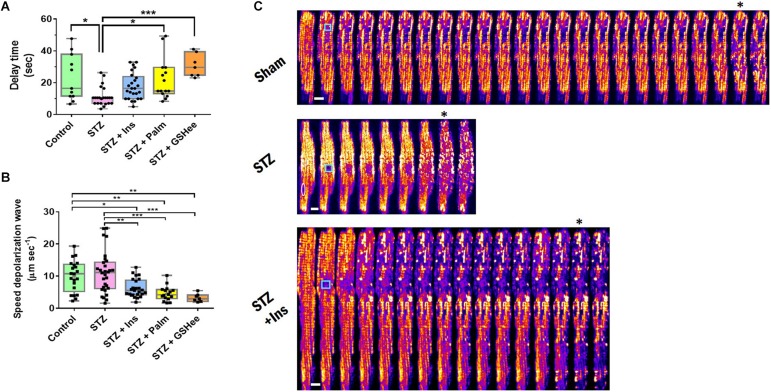
Delay time and speed of propagation of the ΔΨ_m_ depolarization wave in oscillating cardiac myocytes. Freshly isolated cardiomyocytes in Tyrode buffer with 10 mM glucose and 1 mM Ca^2+^ were loaded with TMRM, incubated in the presence of different substrates, and imaged (see Table 1 footnote and section “Materials and Methods”). Depicted are **(A)** delay time (in seconds) before depolarization of the mitochondrial network (≥50% mitochondria) elicited by a laser-flash, and **(B)** speed of propagation of the depolarization wave (in μm/s), exhibited by cardiomyocytes from Sham control, STZ, STZ + Ins, STZ + Palm (STZ preincubated with 0.4 mM palmitate in the presence of 10 mM glucose) and STZ + GSHee (STZ preincubated with 4 mM glutathione ethyl ester in the presence of 10 mM glucose). Sample size: **(A)** Control (Sham), *n* = 11, STZ, *n* = 23 and STZ + Ins, *n* = 25 correspond to 8 experiments/hearts for each group; STZ + Palm, *n* = 15 and STZ + GSHee, *n* = 7 were obtained from four experiments/hearts for each group. **(B)** Control (Sham), *n* = 19, STZ, *n* = 28, and STZ + Ins, *n* = 26, correspond to eight experiments/hearts for each group; STZ + Palm, *n* = 16 and STZ + GSHee, *n* = 7 were obtained from four experiments/hearts for each group. **(C)** Montage of representative examples of Sham, STZ, and STZ + Ins cardiomyocytes from guinea pig heart, showing fast (STZ) or slower (Sham, STZ + Ins) ΔΨ_m_ depolarization propagation after a laser flash (cyan square). In the STZ montage, the white oval delineates the nucleus position. The white bar is equivalent to ∼10 μm. The speed of propagation of depolarization was calculated considering a time resolution of 1.644 s, which corresponds to the acquisition time of each frame (see the section “Materials and Methods” for further details). Significance levels: ^∗^*p* < 0.05; ^∗∗^*p* < 0.01; ^∗∗∗^*p* < 0.001.

*Ventricular cardiac myocytes isolated from hearts of Sham (control), streptozotocin (STZ)-treated (diabetic) and STZ + insulin (STZ + Ins)-treated guinea pigs were assayed for laser flash-induced mitochondrial oscillations as described before (Aon et al., 2003, 2004a). Additionally, Sham control or STZ-cardiomyocytes were preincubated with 4 mM glutathione ethyl ester (GSHee, a cell permeable form of GSH), for at least 2 h or STZ-treated with 0.4 mM palmitate (Palm) in addition to 10 mM glucose in all cases. Palmitate bound to fatty acid-free bovine serum albumin (BSA) (4:1) was prepared as described in Tocchetti et al. (2012, 2015). Controls for potential artifacts introduced by BSA are described in detail elsewhere (Tocchetti et al., 2015). As an example of the data represented in the Table, the cardiac myocytes’ response to the laser flash in the diabetic group was 200/118 meaning that of 200 cells flashed, 118 (or 59%) oscillated in 8 independent experiments/hearts (see also Figure 1 for further characterization of the flash-triggered mitochondrial network oscillations). Oscillating cells, after a localized laser flash, were considered as such when they exhibited at least one complete oscillatory cycle after the first cell-wide depolarization (∼50% of mitochondria depolarized).*

### Network Analysis of ΔΨ_m_ Signal

We applied hand-drawn grid-based analysis as described in Kurz et al. (2010a). Briefly, the first image with a 10% TMRM intensity loss relative to the mean TMRM intensity averaged over 5–10 previous images was defined as the first image of the analyzed stack. In case of movement or instability of the cell during the recording, an Image Stabilizer Plug-in (ImageJ 1.51k, NIH) was used to minimize movement effects. Afterward, averaged pictures of every 10 consecutive images were created and uploaded into Adobe Photoshop CC 2015.5 to construct a hand-drawn grid on a pixel-by-pixel basis, using the original image as a template. Two to four averaged pictures were used for grid generation, representing the beginning, the middle and the end of the recording in order to minimize the impact of small focus shifts. The manually constructed grid was utilized as a template, with each grid mesh representing one mitochondrion. Ensuing wavelet analysis was based on the average pixel intensity given by TMRM fluorescence for each mitochondrion.

### Wavelet Analysis of Oscillatory Frequency From Individual and Mitochondrial Clusters

The spatiotemporal analysis of mitochondrial collective behavior was performed using wavelet analysis as previously described (Kurz et al., 2010a, 2018). In brief, wavelet analysis consists of breaking a signal down into its different scale components, utilizing a wavelet function (“mother wavelet”) (Grossmann and Morlet, 1984). With respect to other frequency evaluation methods such as, e.g., Fourier transform and power spectral analyses, a main advantage of the wavelet method is that it does not need to assume stationarity of the time series (Refinetti, 2004). In addition, our grid-based analysis enables frequency monitoring from an individual as well as collective mitochondrial behavior at any time during the recording, thus providing a precise time-frequency representation. Wavelet analysis was implemented in MATLAB (MathWorks, v8.5.0.197613, R2015a), using the ‘Morlet’ wavelet and performed for each mitochondrion’s TMRM signal. The Morlet wavelet was chosen because of its higher frequency resolution compared to other wavelets and its suitability for the detection of singularities, i.e., discontinuous functions such as mitochondrial oscillations (Kurz et al., 2010a, b).

To simplify and accelerate the calculations, we chose (i) *s*_0_ = 2*dt* as the smallest scale of the wavelet, with *dt* being the amount of time between each value, i.e., the sampling period. Additionally, we (ii) fixed the value of *dj* = 0.25 for the spacing between scales and (iii) the total number of scales *j*_*1*_ was set to $j_{1}=\log_{2}\left(\frac{N}{s_{0}}\right)/d\,j+1$. To define the lower cut-off frequency *f*_*min*_, the TMRM intensity plot of the whole cell was analyzed for its longest period *T* of a synchronized oscillation and set to *f*_*min*_ = 1/(1.1*T*). The upper cut-off frequency, *f*_*max*_, was set to $f_{m\,i\,n}=\frac{1}{4\,d\,t}$. Additionally, we normalized each mitochondrial fluorescence signal by its standard deviation, and the number of time series frames was padded with zeros to the next higher power of 2, thus preventing wraparound from the end of the time series to the beginning and also accelerating the fast Fourier transform that is used in the wavelet transform. For each frame and mitochondrion, we interpolated the power lineplot between *f*_*min*_ and *f*_*max*_ for every scale at a resolution of 0.1 mHz. The frequency at maximum power for the interpolated plot was subsequently determined, and we arrived at a plot of maximal scale frequencies over time for each mitochondrion. We determined the major mitochondrial clusters with similar frequencies for each cardiac myocyte, according to the procedure described in Kurz et al. (2010a, 2018).

### Quantitative Characterization of Mitochondrial Criticality

#### Delay Time

In cardiac myocytes, the proximity of the mitochondrial network to criticality can be evaluated with the delay (or latency) time between the laser flash and the ensuing first cell-wide depolarization of the mitochondrial network, usually comprising at least 50% of the mitochondrial population. We determined the delay time as multiples of the sampling time between two consecutive images (typically either 1.644 s or 3.5 s).

#### Speed of Propagation of the Depolarization Wave

The speed of propagation of the depolarization wave was calculated from the cell wide TMRM signal by determining the time difference Δ*T* between the onset of depolarization and the first maximal depolarization. Dividing the absolute longitudinal length of the cell (in μm) by Δ*T* led to the speed of the depolarization wave in μm/s.

#### Percolation Threshold

For uniformly arranged mitochondria in a plane, percolation theory predicts that at the percolation threshold, *pc*, a spanning cluster exists, comprising ∼60% of the mitochondrial network. This network will form throughout the cell to initiate synchronized mitochondrial depolarizations after a perturbation (Aon et al., 2004a). Thus, at *p*_c_, a “critical” mass of mitochondria surpassing a certain level of oxidative stress will initiate, even after a small perturbation, a transition of the network into a pathophysiological state of cell-wide synchronized ΔΨ_m_ oscillations. Under these conditions, a local event (e.g., a mitochondrial depolarization) may propagate globally throughout the cell since at *pc* the probability for a single mitochondrion to belong to the largest (spanning) cluster increases dramatically (Aon et al., 2004a).

The percolation threshold *p*_c_ was considered as the percentage of depolarized mitochondria, at maximal first depolarization (at least 50% of the mitochondrial population), with respect to the total number of mitochondria in the grid. We estimated *pc* by taking advantage of the fact that percolation processes at *pc* are organized as fractals (Aon et al., 2004a, b); at *pc*, the mass of the spanning cluster increases with the size of the lattice, *L*, as a power law, *L*^Df^, with *D*_f_ as the fractal dimension. Assuming that mitochondria are arranged in a quasi-square lattice, L^2^, here, *L* corresponds to L = √ (the total number of mitochondria as obtained from our grid-based analysis), and *D*_f_ = 1.82 (Aon et al., 2004b); both values allow an estimation of *pc* for each myocyte. This result was then tested by quantitatively evaluating the fraction of polarized/depolarized mitochondria in two different ways. In the first approach, we superimposed the created grid and the frame in which the first depolarization is maximally distinct. Using the cell-counter plug-in in ImageJ, polarized mitochondria were marked and subtracted from the total number of mitochondria in the grid. The remaining mitochondria were considered depolarized and their amount was divided by the total number of mitochondria, thus leading to the percolation threshold.

The second approach was numeric. A cutoff value for mitochondrial TMRM fluorescence, to evaluate their energetic status as “polarized” or “depolarized,” was determined from the distribution of the normalized fluorescence intensity of every individual mitochondrion with respect to its respective averaged TMRM fluorescence baseline level before depolarization. A mitochondrion with a fluorescence intensity loss of at least 25% with respect to its baseline intensity value was considered as “depolarized,” thus belonging to the “spanning cluster.” Finally, we compared our results from the first and second approach and found similar results with differences not larger than ±1.5% in each group. We therefore validated our first approach.

### Statistics

The wavelet analysis and fitting routines were performed with Matlab (MathWorks, v8.5.0.197613, R2015a). Other statistical analyses were performed using version 94E of OriginPro 2017 and Excel (2016). The results are presented as mean ± SEM/SD and the statistical significance of the differences between groups/treatments was evaluated by one-way ANOVA. Outliers in Figures 1A,B were excluded from the statistics when above or below 1.5 times the interquartile range comprised between the 75 and 25% percentiles, respectively (Aitken et al., 2010).

## Results

### Cardiomyocytes From T1DM GPs Are More Prone to Exhibit Cell-Wide Synchronized Oscillations

It is well-known that the mitochondrial cardiac network exhibits a higher propensity to the triggering of mitochondrial oscillations under oxidative stress, a condition prevalent not only in acute heart failure (Goh et al., 2016) but also in the chronic diabetic state (Aon et al., 2009, 2015). Using laser flash-triggered ΔΨ_m_ oscillations, we tested whether the induction of T1DM by STZ treatment renders cardiac cells more prone to exhibit mitochondrial oscillations. As shown in Table 1, diabetic cardiac myocytes were about twice as vulnerable to laser flash-triggered oscillations (118 out of 200 flashed STZ cardiomyocytes oscillated, corresponding to 59%) as compared to Sham controls (32%). Insulin treatment of diabetic GPs brought vulnerability back close to control levels (36%).

The oxidative stress dependence of diabetic cardiomyocytes’ susceptibility to oscillate was further examined by preincubation for at least 2 h with 4 mM GSHee in the presence of 10 mM glucose. Indeed, GSHee was able to increase the intracellular levels of glutathione (Supplementary Figure S1) and decrease to control levels the percentage of cardiac cells susceptible to oscillate after a laser flash whereas it had no effect on Sham controls. Palmitate (Palm) addition, in the presence of glucose, improves the redox status of diabetic cardiac myocytes, decreasing ROS levels (Tocchetti et al., 2015). Under these conditions, 0.4 mM Palm diminished the propensity to oscillate of diabetic heart cells to control levels (33%) (Table 1). As a caveat, the ∼30% susceptibility to laser flash-triggered oscillations corresponds, independently from any kind of treatment, to the baseline susceptibility value likely due to the oxidative stress elicited by the isolation procedure itself.

Taken together, the data presented show that cardiac myocytes isolated from diabetic GPs exhibit the highest vulnerability to laser flash-triggered oscillations, a condition from which they can be rescued to Sham control levels by insulin treatment of STZ-treated GPs. Preincubation with GSHee or Palm in the presence of glucose also reduces the propensity of diabetic cardiac myocytes to oscillate when compared with Sham control levels.

### The Mitochondrial Network of T1DM Cardiomyocytes Is Closer to Criticality

The above results suggest that the mitochondrial network of diabetic cardiac myocytes is closer to mitochondrial criticality than non-diabetic cardiac myocytes. To quantitatively assess the degree of the mitochondrial network’s proximity to criticality, we quantified the delay (or latency) time as the interval comprised between the laser flash until the first cell-wide mitochondrial depolarization (> ∼ 50% of the mitochondria).

As shown in Figure 1A, the time delay was significantly shorter in the diabetic group as compared to controls (23 ± 4 s *vs.* 11 ± 1 s, *p* = 0.011), while insulin treatment as well as Palm and GSHee increased the latency period of the mitochondrial network to depolarize after the laser flash (18 ± 2 s; 22 ± 3 s; 31 ± 3 s, respectively) thus bringing the mitochondria’s susceptibility to control levels. A twofold decrease in the delay time of T1DM cardiac myocytes to trigger laser flash-elicited oscillations with respect to controls (Figure 1A) agrees very well with the two-fold increase in the propensity of diabetic cells to exhibit mitochondrial oscillations (Table 1).

Furthermore, we found that the p_c_ for the control group was at 60 ± 0.02% (*n* = 14), in agreement with previous results (Aon et al., 2004a), whereas the p_c_ for diabetic animals was significantly elevated (76 ± 0.02%, *n* = 19). Importantly, insulin treatment, Palm or GSHee rescued p_c_ close to or at control levels: 66 ± 0.03% (*n* = 6), 60 ± 0.019% (*n* = 6), and 57 ± 0.03% (*n* = 3), respectively (Table 2). These findings are in agreement with the interpretation that formation of synchronized clusters of depolarizing mitochondria in cardiomyocytes from T1DM animals involves a larger critical cohort of mitochondria, suggesting a more pronounced inter-mitochondrial coupling mechanism (Kurz et al., 2015). Insulin treatment disrupts this process, reducing p_c_, as do Palm and GSHee preincubated cells.

**TABLE 2:** Percolation threshold (p_c_).

Group	Measured (%±SEM)	Estimated
Sham	60 ± 0.022	57%
STZ	76 ± 0.018	56%
STZ + Ins	66 ± 0.034	58%
STZ + Palm	60 ± 0.019	57%
STZ + GSHee	57 ± 0.032	56%

*Sample size for measured and estimated percolation threshold for: Sham (*n* = 14), STZ (*n* = 19), STZ + Ins (*n* = 6), STZ + Palm (*n* = 6), STZ + GSH (*n* = 3).*

Next, we analyzed the speed of propagation of depolarization throughout the cardiac myocyte, once the mitochondrial network transitions into cell-wide synchronized ΔΨ_m_ oscillations. Interestingly, the speed of the depolarization waves in control and T1DM cardiomyocytes were similar with 9.5 ± 1.08 μm/s and 11.5 ± 1.2 μm/s, respectively. However, we found a significant decrease in the depolarization speed in T1DM GPs treated with insulin (6.2 ± 0.56 μm/s) or in cardiac myocytes isolated from this group that were preincubated with either 0.4 mM Palm (4.6 ± 0.57 μm/s) or 4 mM GSHee (3.2 ± 0.48 μm/s) in the presence of 10 mM glucose. Palm and GSHee also produced a significant decrease of the depolarization wave speed when compared with the control group, although to a lesser degree (Figure 1B). Representative montages of laser flashed cardiac myocytes are shown in Figure 1C for fast and slow wave propagation, respectively.

In aggregate, the data presented show that the propensity of the mitochondrial network of diabetic cardiomyocytes, as measured by susceptibility to oscillate (Table 1) or latency period after the laser flash (Figure 1A), is at least twofold higher than in non-diabetic or diabetic animals treated with insulin. Importantly, acute pretreatment interventions known to improve the redox status of cardiac cells such as Palm or GSHee (Tocchetti et al., 2012, 2015; Aon et al., 2015; Bhatt et al., 2015; Cortassa et al., 2018) protect the cells, distancing them from oxidative stress conditions leading to criticality.

### Wavelet Analysis of Mitochondrial Network Dynamics

To further examine and characterize the spatiotemporal dynamic behavior of the mitochondrial networks, we utilized grid-based wavelet analysis of cell fluorescence obtained by imaging with two-photon laser scanning microscopy.

The grid-based wavelet approach enables monitoring of the time-dependent evolution of ΔΨ_m_ oscillatory frequencies from mitochondria at both single and population levels, without the need to assume stationarity of the time series (Supplementary Figures S2–S4; for Sham, STZ, and STZ + Ins, respectively) (Kurz et al., 2010a, 2017). Using an average TMRM-fluorescence image of the cell as a template (Supplementary Figure S2A, center), we created a hand-drawn grid (Supplementary Figure S2B, center). This allowed us to identify and label every single mitochondrion thus enabling their collective (Supplementary Figure S2A, left panel) or individual (Supplementary Figure S2B, right panel) monitoring over time. Using grid-based wavelet analysis we were able to determine the temporal evolution of mitochondrial frequency, as well as to differentiate clustered (Supplementary Figure S2B, top panel) from non-clustered (Supplementary Figure S2B, bottom panel) mitochondria. The wavelet transform provides a temporally resolved frequency representation of the TMRM signal over time for the whole mitochondrial population, which dropped from ∼40 to 20 mHz during the recording (Supplementary Figure S2A, left panel).

The largest group of mitochondria with similar frequencies for a specific point in time constitutes a cluster exhibiting highly correlated and coherent ΔΨ_m_ signal, involved in the macroscopic, cell-wide oscillations (Kurz et al., 2017). The size of the cluster influences synchronization; large clusters take longer to synchronize, thus their common frequency is lower compared to smaller clusters (Kurz et al., 2017).

**FIGURE 2:**
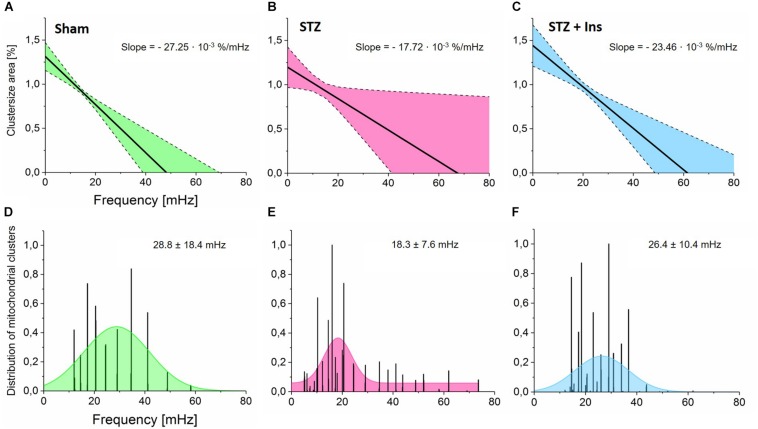
Wavelet analyses of mitochondrial networks and the relationship between cluster size, frequency and frequency distribution in mitochondrial clusters. Isolated cardiomyocytes from Sham, STZ and STZ + Ins groups were imaged and analyzed as described in the legend of Figure 1 (see the section “Materials and Methods”). **(A–C)** Cluster area normalized by the whole myocyte area as a function of frequency in cardiomyocytes isolated from **(A)** Control (Sham, n = 8; green), **(B)** Diabetic (STZ, *n* = 9, pink) or **(C)** Diabetic treated with insulin (STZ + Ins, *n* = 6, blue) guinea pig hearts. Notice the weaker dependence of frequency on cluster size exhibited by diabetic as compared to controls (∼35% decrease in slope) **(A,B)** whereas insulin treatment of diabetic guinea pig rescues the effect of diabetes to control levels as can be judged by the similar slope of the cluster size *vs.* frequency relationship **(C)**. **(D–F)** Frequency distribution exhibited by mitochondrial clusters across all myocytes analyzed in the three groups. Notice that the mean of the frequency distribution in Sham control cardiomyocytes is lower than in diabetic ones which display a widespread distribution, suggesting greater dynamic heterogeneity in mitochondrial clusters from diabetic cardiac myocytes.

The relationship between cluster size and the behavior of mitochondrial frequency at every time point during the recording was analyzed. The strength of the relationship between cluster size *vs.* frequency is measured by the slope, which in cardiomyocytes from diabetic animals was found as −17.72⋅10^−3^%/mHz, which is ∼ 35% smaller than in controls (−27.25⋅10^−3^%/mHz), see Figures 2A,B. Importantly, cardiac myocytes from the insulin-treated diabetic GPs exhibited a similar slope (−23.46⋅10^−3^%/mHz) as compared to control cells (Figures 2A,C), thus suggesting that insulin rescues the T1DM mitochondrial network behavior to control levels. Present and previous data obtained on T1DM cardiomyocytes showing that insulin treatment as well as redox-boosting interventions such as GSHee or Palm preincubation improve the redox status of diabetic GPs (Tocchetti et al., 2015), are in agreement with their protective effects from mitochondrial criticality (Figure 1).

Next, we analyzed the mitochondrial frequency distribution as a function of time. From the combined frequency histograms (within 0 to 80 mHz range) of the mitochondrial population for every recorded image, we obtained a Gaussian fit function to determine the frequency bandwidth (mean ± SD) for the control as (28.8 ± 18.4) mHz (Figure 2D). According to Supplementary Figure S2A, this suggests that the cluster size area in the control group represents approximately 53% of the whole cell volume. When subtracting the area comprised of the cell nuclei (∼10% of the cell volume), the primary cluster in Sham cells represents ∼59% of the mitochondrial population. This value agrees very well with the estimated size of the spanning cluster according to percolation theory (Aon et al., 2004a). Furthermore, the mean frequency in diabetic cells was lower with 18.3 ± 7.6 mHz (Figure 2E) than in control cells. This result indicates the presence of large clusters with predominant low-frequency, high-amplitude oscillations, likely resulting from relatively slow rate-controlling steps in the synchronization process (Kurz et al., 2010b, 2017). Interestingly, insulin treatment restores the frequency distribution to almost control levels, exhibiting a frequency bandwidth of (26.4 ± 10.4) mHz (Figure 2F).

Overall, the wavelet analysis demonstrates that the oscillatory frequency distribution exhibited by mitochondria is inversely correlated with cluster size in both control and diabetic animals, confirming previous findings for non-diabetic animals (Kurz et al., 2010a, 2015). Diabetic cardiac myocytes exhibit large mitochondrial clusters with predominant bandwidth distribution around low-frequency values, corresponding to long-period, high-amplitude oscillations. However, the dynamics of frequency resulting from the collective behavior of mitochondria doubles as a function of time, as revealed by their tendency to oscillate with shorter periods.

### *In vivo* Echocardiographic Assessment of Heart Function in Type 1 Diabetic, STZ-Treated Guinea Pigs (T1DM)

To further investigate whether cardiac dysfunction exists in this T1DM GP animal model, we assessed *in vivo* cardiac function in the three experimental groups: Sham, and diabetic (STZ) without or with insulin treatment (STZ + Ins). Using echocardiographic measurements in conscious guinea pigs, we sought to determine whether cardiac function was affected by the STZ treatment, and if it could be rescued by insulin treatment.

**FIGURE 3:**
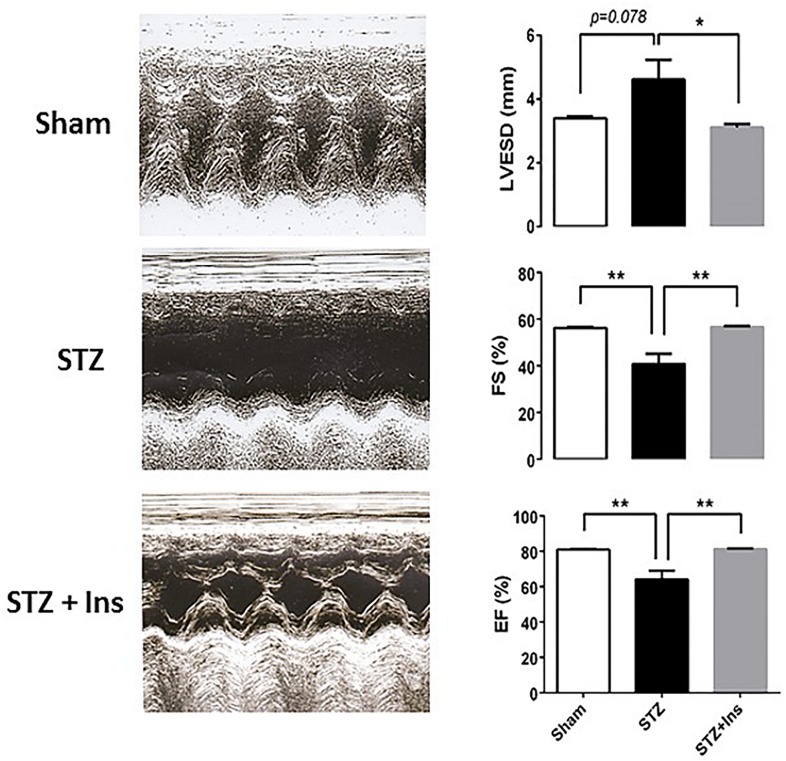
Echocardiographic measurements in guinea pig control (Sham) and diabetic without (STZ) or with insulin treatment (STZ + Ins). Left panels show representative *trans*-thoracic echocardiograms from Sham, STZ and STZ + Ins performed in conscious guinea pig using the Sequoia Acuson C256 (Malvern, PA) system, equipped with a 15 MHz linear transducer respectively. The three panels on the right display the statistical comparison of the significantly different functional variables: LVESD, FS and EF exhibited by the three groups [see also Table 3]. *p < 0.05; **p < 0.01. M-mode echocardiogram was obtained in the parasternal short axes view of the left ventricle [LV] at the level of the papillary muscles and at sweep speed of 200 mm/s. From left ventricular end diastolic [LVEDD] and end systolic dimension [LVESD], the percent left ventricular contractility, estimated by the fractional shortening [FS] and the ejection fraction [EF], was obtained according to the following equation: FS (%) = [(LVEDD – LVESD)/LVEDD] X 100 The left ventricular mass [LV mass] was derived and used in the assessment of left ventricular hypertrophy and enlargement, using the following equation: LV mass (mg): 1.055[(IVSD + LVEDD + PWTED) ^3^ – (LVEDD) ^3^] where 1.055 is the specific gravity of the myocardium, IVSD is the inter-ventricular septal thickness at end diastole and PWTED is the posterior wall thickness at end diastole. LVEDD, left ventricle end diastolic diameter; LVESD, left ventricle end systolic diameter; PWTED, posterior wall thickness at end diastole; IVSD, inter-ventricular septal thickness at end diastole; FS, fractional shortening; EF, ejection fraction; LV mass, left ventricular mass; RWT, relative wall thickness; and HR, heart rate. Data expressed in mean ± SEM.

The results depicted in Figure 3 show that both, fractional shortening [FS] and ejection fraction [EF] (mid and bottom panels, respectively) are significantly decreased in the T1DM (STZ) group as compared to Sham and STZ + Ins, and that the left ventricle end systolic diameter [LVESD] (top panel) from STZ hearts is larger than in Sham and STZ + Ins. Importantly, insulin therapy was able to rescue LV function to Sham levels in the STZ heart (Figure 3). Heart rate was not different among the three animal groups (see Table 3). All measurements and their statistical analysis are displayed in Table 3.

**TABLE 3:** Echocardiographic measurements for cardiac morphology and function of guinea pig control (Sham) and diabetic without (STZ) or with insulin treatment (STZ + Ins).

	Sham	STZ	STZ + Ins
LVEDD (mm)	7.77 ± 0.06	7.63 ± 0.52	7.20 ± 0.23
LVESD (mm)	3.40 ± 0.07	4.62 ± 0.62	3.12 ± 0.10*
IVSD (mm)	2.23 ± 0.03	2.02 ± 0.07	2.15 ± 0.06
PWTED (mm)	2.05 ± 0.06	1.98 ± 0.07	2.07 ± 0.06
FS (%)	56.2 ± 0.602**	40.9 ± 4.35	56.7 ± 0.401**
EF (%)	80.8 ± 0.522**	64.1 ± 5.03	81.2 ± 0.348**
LV mass (mM)	1379 ± 40	1219 ± 99	1209 ± 90
RWT	0.53 ± 0.01	0.54 ± 0.05	0.58 ± 0.01
HR (b/min)	382 ± 20	387 ± 22	393 ± 8

Guinea pigs from the Sham and STZ group were tested after 4 weeks of vehicle [Sham; *n* = 6] or streptozotocin [STZ; *n* = 6] injection. The STZ group treated with insulin [STZ + Ins; *n* = 6] was monitored after 6 weeks that included two additional weeks of insulin treatment according to the experimental protocol described in Tocchetti et al. (2015). Highlighted are the variables that exhibited significant differences among groups, with respect to STZ (see Figure 3). *p < 0.05; **p < 0.01. LVEDD, Left ventricle end diastolic diameter; LVESD, left ventricle end systolic diameter; PWTED, posterior wall thickness at end diastole; IVSD, inter-ventricular septal thickness at end diastole; FS, fractional shortening; EF, ejection fraction; LV mass, left ventricular mass; RWT, relative wall thickness; and HR, heart rate. Data expressed in mean ± SEM.

Together, the data obtained show LV pump dysfunction in T1DM (STZ) hearts, an effect that can be reversed by insulin treatment.

## Discussion

The main contribution of this work is to show that type 1 diabetes, a chronic metabolic disorder, may favor the transition of the mitochondrial cardiac network to criticality, increasing the likelihood of cell-wide depolarization followed by mitochondrial oscillations. We show that the mitochondrial network from T1DM cardiac myocytes exhibits a twofold higher propensity to oscillate, in agreement with a delay time between laser flash and depolarization that is twice as short. Importantly, insulin treatment of diabetic GPs is shown to rescue cardiac myocytes’ behavior to control levels. Wavelet analysis further reveals that in T1DM cardiac cells, the collective dynamic response of mitochondria exhibits distinctive features consistent with organized behavior around larger clusters with frequency lock at lower values, and an overall weaker dependence of frequency on cluster size, as compared to controls. Overall, the data obtained agrees with the idea that the degree of oxidative stress defines mitochondrial criticality and its unfolding, i.e., shorter latency period until the first depolarization without affecting the speed of the depolarization wave which, after the triggering, can instead be significantly slowed by interventions improving the cellular redox environment. The wavelet analysis further supports this interpretation by showing that redox boosting and insulin interventions re-increase the strength of the relationship between cluster size *vs.* frequency, indicating the reappearance of smaller clusters with higher frequencies (Figures 2A–F). These dynamics may result from ROS-dependent inter-mitochondrial coupling mechanisms affecting the synchronization process (Kurz et al., 2015).

A stronger inverse relation between cluster size and frequency as well as increasingly large frequency distributions are signatures of spatio-temporal heterogeneity and the presence of dynamic mitochondrial clusters (Kurz et al., 2010a, 2017). In agreement with previous results (Kurz et al., 2010a, 2015), the inverse relationship between cluster-size and frequency found implies that the larger a cluster a mitochondrion belongs, the lower the frequency (i.e., the longer the period) of its oscillatory cycles, suggesting clustering around a few main (low) frequencies. The fragmentation of the mitochondrial network into smaller clusters with a combined lower synchronicity reflects mitochondrial adaptation to altered substrate/redox conditions (Kurz et al., 2015).

Our initial hypothesis, i.e., that cardiac cells from diabetic GPs would be closer to criticality because of their higher oxidative stress, is fully supported by the results of the present work. Indeed, T1DM mitochondria and cardiac myocytes display an oxidative environment (Tocchetti et al., 2015), in agreement with their higher propensity to exhibit laser flash-induced mitochondrial oscillations. According to the results of the present work, the prevalent oxidizing conditions favored by the chronic diabetic state (Tocchetti et al., 2012, 2015; Bhatt et al., 2015; reviewed in Aon et al., 2015) or by reperfusion after ischemic injury under acute heart failure conditions (Akar et al., 2005; Brown et al., 2010; Zhou et al., 2014; Solhjoo and O’Rourke, 2015) can both increase the susceptibility of the cardiac mitochondrial network to trigger mitochondrial oscillations leading to arrhythmias.

### Mitochondrial Criticality at the Origin of Cardiac Arrhythmias

Previous work has shown that conditions leading to mitochondrial criticality set the stage for catastrophic arrhythmias (Akar et al., 2005; Brown et al., 2010; Zhou et al., 2014). Mitochondrial criticality appears as a seminal event followed by escalating adverse consequences, from the mitochondrion to the whole cell and organ levels, producing electrophysiological and contractile dysfunction associated with reperfusion after ischemic injury (Aon et al., 2003, 2006; Akar et al., 2005) and likely in heart failure (Dey et al., 2018). Central to this chain of adverse events is the oxidation of the cytoplasmic GSH pool which triggers mitochondrial depolarization and NAD(P)H oxidation (Aon et al., 2007; Slodzinski et al., 2008; Brown et al., 2010; Xie et al., 2013), thereby fueling mitochondrial ROS-induced ROS release (Zorov et al., 2000; Aon et al., 2003). Consistent with previous work, treatments known to improve the redox condition of cardiac cells, protected diabetic cardiac myocytes from reaching criticality by either increasing their antioxidant capacity (insulin, GSHee) or decreasing ROS levels (Palm), as revealed by their elicited increase in the latency period after the laser flash (Figure 1A), the decrease in the speed of the depolarization wave (Figure 1B), or the recovery of their collective dynamic behavior as unveiled by wavelet analysis (Figure 2).

The dynamic behavior of the network’s response during oscillations also presented distinctive features in T1DM cells when compared to Sham controls. Although diabetic cardiac myocytes can depolarize and oscillate twice as fast as controls, both groups exhibit similar speeds of propagation of the depolarization wave. This result implies that the proximity to mitochondrial criticality (as measured by the delay time) is determined by the level of initial oxidative stress, i.e., the higher the stress the lower the delay time. This effect likely underlies the p_c_ increase in the diabetic state (Table 2) which appears to be mainly determined by the enhanced oxidative stress exhibited by the cardiomyocytes from diabetic animals, resulting in increased number of mitochondria attaining threshold levels of oxidative stress, a necessary condition to belong to the critical mitochondrial cluster (Aon et al., 2003, 2004a; Kurz et al., 2010a). This interpretation is consistent with the sensitivity of mitochondrial collective dynamics to the laser flash perturbation, as revealed by the latency period, and to redox boosting (GSHee, Palm) and insulin interventions (Figures 1A,B). However, perturbations in the mitochondrial network topology may also have an influence on mitochondrial dynamics, e.g., as shown in heart failure (Goh et al., 2016).

Moreover, irrespective of the fact that cardiac myocytes have reached criticality, interventions leading to the improvement of the cellular redox environment can alter the subsequent unfolding of the process by significantly slowing the speed of propagation of the depolarization wave, and increasing the delay time (Figures 1A,B). These results and their interpretation are consistent with our previous work (Aon et al., 2003, 2004a; Kurz et al., 2010a), as well as a wealth of reported evidence on the protective effects of favorable redox on mitochondrial function, and their potential cardioprotective consequences (Tocchetti et al., 2012, 2015; Aon et al., 2015; Bhatt et al., 2015; Cortassa et al., 2018). In contrast, unfavorable redox conditions lead to mitochondrial dysfunction (Aon et al., 2007; Cortassa et al., 2014) along with impairment of contractile performance associated with diabetic cardiomyopathy (Tocchetti et al., 2012, 2015; Bhatt et al., 2015; Schilling, 2015) and arrhythmias (Aon et al., 2009; Jeong et al., 2012; Aggarwal and Makielski, 2013; Xie et al., 2013; Kembro et al., 2014; Zhou et al., 2014; Galloway and Yoon, 2015; Solhjoo and O’Rourke, 2015).

### The T1DM GP Animal Model and Its Potential Role for Investigating Cardiac Arrhythmias

As described in the current and previous investigation (Xie et al., 2013; Tocchetti et al., 2015; Cortassa et al., 2017), T1DM GPs harbor glucose levels similar to those found in human T1DM, and exhibit contractile dysfunction, as indicated by the impaired FS and EF (Figure 3). The chronic administration of insulin rescued LV function in T1DM GPs *in vivo* (Figure 3) and shielded isolated cardiac myocytes and mitochondria from oxidative stress as suggested by the decreased ROS emission while improving the mitochondrial energetic response. Nevertheless it failed to forestall contractile and relaxation impairment in T1DM myocytes challenged with both high glucose and the β1/β2 agonist, isoproterenol (Tocchetti et al., 2015). This inability could stem from a direct negative impact of insulin on the adrenergic signaling pathway, thus affecting the ISO-elicitable inotropic reserve of the cardiomyocyte (Fu et al., 2014). This possibility warrants further, in-depth investigation.

Relevant to the present study is the finding that T1DM GPs hearts showed higher vulnerability to arrhythmias when GSH was oxidized by diamide treatment, enhancing action potential duration (APD) heterogeneity and the arrhythmia scoring index (Xie et al., 2013). This evidences lends further support to the notion that APD heterogeneity underlies the higher propensity to arrhythmias exhibited by the T1DM heart (Solhjoo and O’Rourke, 2015). At the same time it reiterates the critical role played by the redox state of the glutathione pool in keeping the mitochondrial network apart from criticality (Figures 1A,B) (Aon et al., 2007; Slodzinski et al., 2008), thus in suppressing arrhythmias (Brown et al., 2010).

Although cardiomyocytes isolated from T1DM hearts subjected to chronic insulin treatment were less prone to exhibit criticality (Figures 1, 2), normalization of glycemic levels by insulin did not reverse the proarrhythmic properties in T1DM hearts subjected to diamide treatment (Xie et al., 2013). This apparent discrepancy could be explained by the eventuality that triggering of arrhythmias is likely more dependent upon the overall degree of mitochondria membrane potential polarization in the cellular network than in individual cells. Supporting this possibility is the apparent key role played by APD heterogeneity in arrhythmias’ propensity that, in turn, results from the coupling status between the mitochondrial energy state and electrical excitability mediated by the sarcolemmal K_ATP_ current (Solhjoo and O’Rourke, 2015).

## Conclusion

Overall, the results of the present work expand on the potential role of mitochondrial criticality in the chronic setting of a metabolic disorder such as T1DM. Our results also suggest that the higher susceptibility to mitochondrial criticality exhibited by T1DM cardiac myocytes can be at least partly prevented by insulin therapy or interventions leading to the improvement of the cellular redox status.

## Data Availability Statement

The datasets generated for this study are available on request to the corresponding author.

## Ethics Statement

The animal study was reviewed and approved by Institutional Animal Care/Use Committee, Johns Hopkins University, Baltimore, MD, United States.

## Author Contributions

MA and FK conceived the study. DB and MA performed echocardiographic measurements. DB, MA, and NP analyzed and interpreted the echocardiographic data. MA and LV performed microscopy experiments. FK provided analytical tools. LV, SC, MA, JJ, and FK analyzed the data. LV, SC, BO’R, AA, MB, NP, SS, MA, and FK interpreted experiments and wrote the manuscript.

## Conflict of Interest

The authors declare that the research was conducted in the absence of any commercial or financial relationships that could be construed as a potential conflict of interest.

## Supplementary Material

FIGURE S1 STZ cardiomyocytes preincubated with GSHee and loaded with MCB. Freshly isolated cardiomyocytes from STZ-treated GPs were preincubated with 4 mM of the cell permeable GSH ethyl ester (GSHee) for at least 2 h, loaded with 50 μM of the GSH fluorescent reporter monochlorobimane (MCB) and imaged with two photon microscopy (Cortassa et al., 2004). A significant increase of the intracellular GSH levels is observed in STZ myocytes preincubated with GSHee compared to non-preincubated controls (*n* = 33; 3 hearts). The decrease in the susceptibility to flash-triggered oscillations by T1DM (STZ) cardiomyocytes preincubated with GSHee can be explained by enhanced GSH intracellular pool that diminishes oxidative stress. ****p < 0.0001.

FIGURE S2 Wavelet analysis of the mitochondrial network of ventricular myocytes isolated from diabetic guinea pig heart. Two-photon laser scanning fluorescence imaging of cardiomyocytes loaded with 100 nM tetramethyl rhodamine methyl ester (TMRM) was performed as described elsewhere (Aon et al., 2003, 2007). Using a cardiac myocyte fluorescent image as a template, a hand-drawn grid was created with one mitochondrion in each raster element. Using this grid, the temporal properties of individual mitochondria as well as the whole network can be analyzed enabling the distinction between clustered (green outline) *vs.* non-clustered mitochondria (red outline). Depicted is the mitochondrial network of a sham control cell (center left), and its respective grid with each element corresponding to individual mitochondria (center right). The absolute squared wavelet transform over frequency and time of the TMRM signal of the whole cardiomyocyte (**A**, left panel) or individual mitochondria **(B)** belonging to the spanning cluster (**B**, top panel) or not (**B**, bottom panel), are represented. Notice the drop from about ∼40 to 20 mHz in the major frequency exhibited by the mitochondrial network.

FIGURE S3 Wavelet analysis in a diabetic ventricular myocyte (STZ). Depicted is the mitochondrial network of a STZ cell (center left), and its respective grid with each element corresponding to individual mitochondria (center right). The absolute squared wavelet transform over frequency and time of the TMRM signal of the whole cardiomyocyte (**A**, left panel) or individual mitochondria **(B)** belonging to the spanning cluster (**B**, top panel) or not (**B**, bottom panel), are represented. There are higher frequencies up to ∼80 mHz of the spanning cluster when compared with the wavelet analysis of the control (sham) cell (Supplementary Figure S2). This is in agreement with the analysis in Figure 2.

FIGURE S4 Wavelet analysis in a diabetic ventricular myocyte after insulin treatment (STZ + Ins). Mitochondrial network of a STZ + Ins cell (center left), and its respective grid with each element corresponding to individual mitochondria (center right). The absolute squared wavelet transform over frequency and time of the TMRM signal of the whole cardiomyocyte (**A**, left panel) or individual mitochondria **(B)** belonging to the spanning cluster (**B**, top panel) or not (**B**, bottom panel), are represented. There are mostly lower frequencies ∼20 mHz of the spanning cluster when compared with the wavelet analysis of the STZ cell (Supplementary Figure S3). This is in agreement with the analysis in Figure 2.
