# Supplementary figures and images for: Diabetes Increases the Vulnerability of the Cardiac Mitochondrial Network to Criticality
Source: Front Physiol. 2020 Mar 10;11:175. doi: 10.3389/fphys.2020.00175 (PMC7077512; doi:10.3389/fphys.2020.00175)

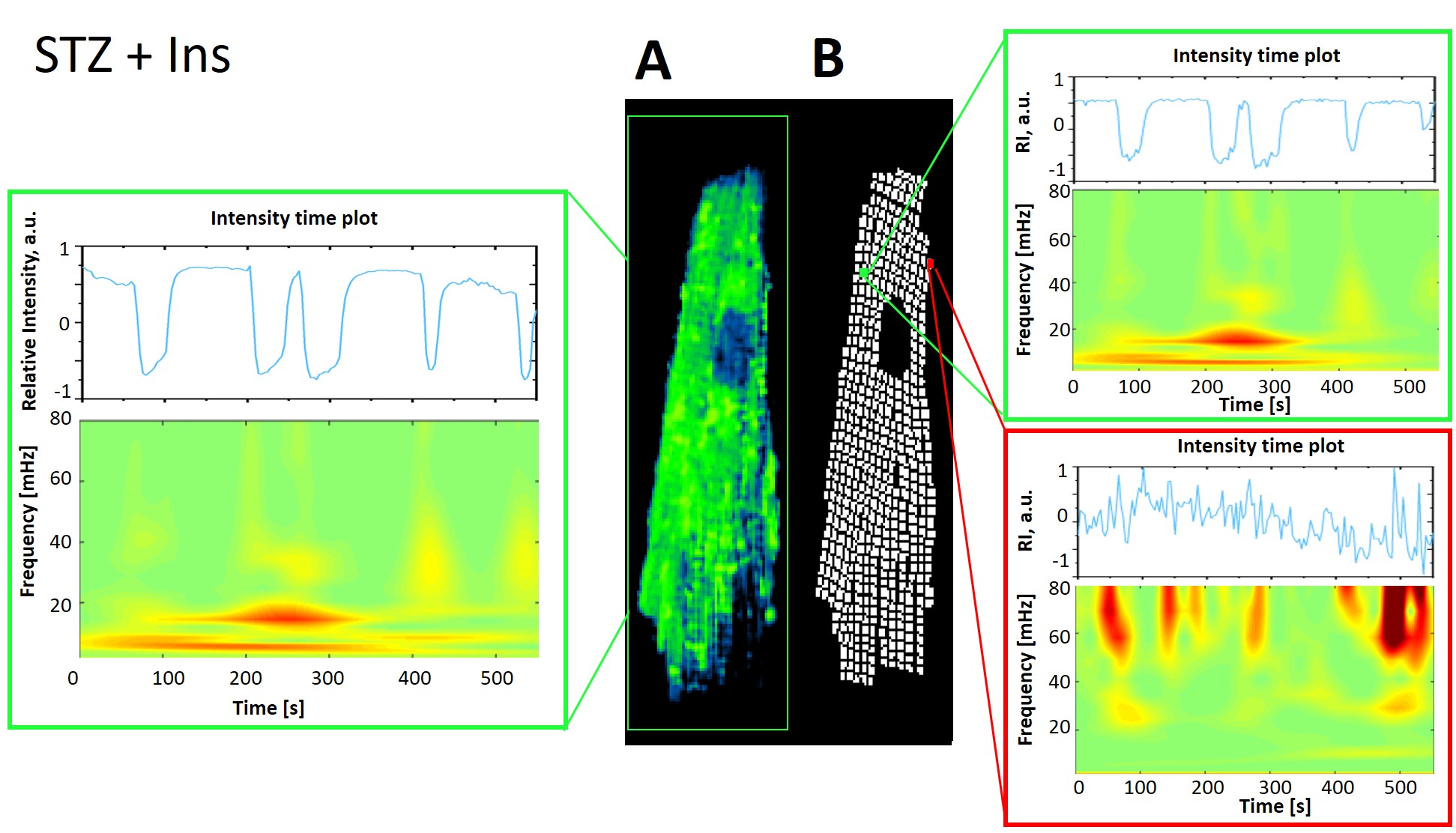

Supplement: FIGURE S1 — STZ cardiomyocytes preincubated with GSHee and loaded with MCB. Freshly isolated cardiomyocytes from STZ-treated GPs were preincubated with 4 mM of the cell permeable GSH ethyl ester (GSHee) for at least 2 h, loaded with 50 μM of the GSH fluorescent reporter monochlorobimane (MCB) and imaged with two photon microscopy (Cortassa et al., 2004). A significant increase of the intracellular GSH levels is observed in STZ myocytes preincubated with GSHee compared to non-preincubated controls (n = 33; 3 hearts). The decrease in the susceptibility to flash-triggered oscillations by T1DM (STZ) cardiomyocytes preincubated with GSHee can be explained by enhanced GSH intracellular pool that diminishes oxidative stress. ****p < 0.0001. [file Image_1.jpeg]

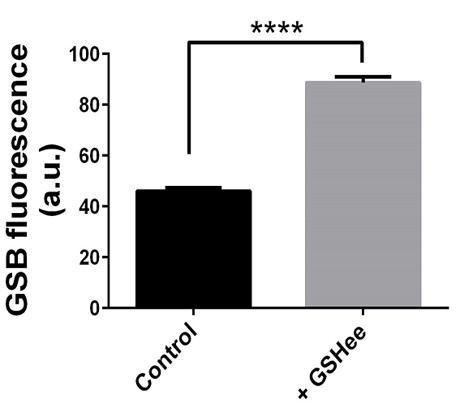

Supplement: FIGURE S2 — Wavelet analysis of the mitochondrial network of ventricular myocytes isolated from diabetic guinea pig heart. Two-photon laser scanning fluorescence imaging of cardiomyocytes loaded with 100 nM tetramethyl rhodamine methyl ester (TMRM) was performed as described elsewhere (Aon et al., 2003, 2007). Using a cardiac myocyte fluorescent image as a template, a hand-drawn grid was created with one mitochondrion in each raster element. Using this grid, the temporal properties of individual mitochondria as well as the whole network can be analyzed enabling the distinction between clustered (green outline) vs. non-clustered mitochondria (red outline). Depicted is the mitochondrial network of a sham control cell (center left), and its respective grid with each element corresponding to individual mitochondria (center right). The absolute squared wavelet transform over frequency and time of the TMRM signal of the whole cardiomyocyte (A, left panel) or individual mitochondria (B) belonging to the spanning cluster (B, top panel) or not (B, bottom panel), are represented. Notice the drop from about ∼40 to 20 mHz in the major frequency exhibited by the mitochondrial network. [file Image_2.jpeg]

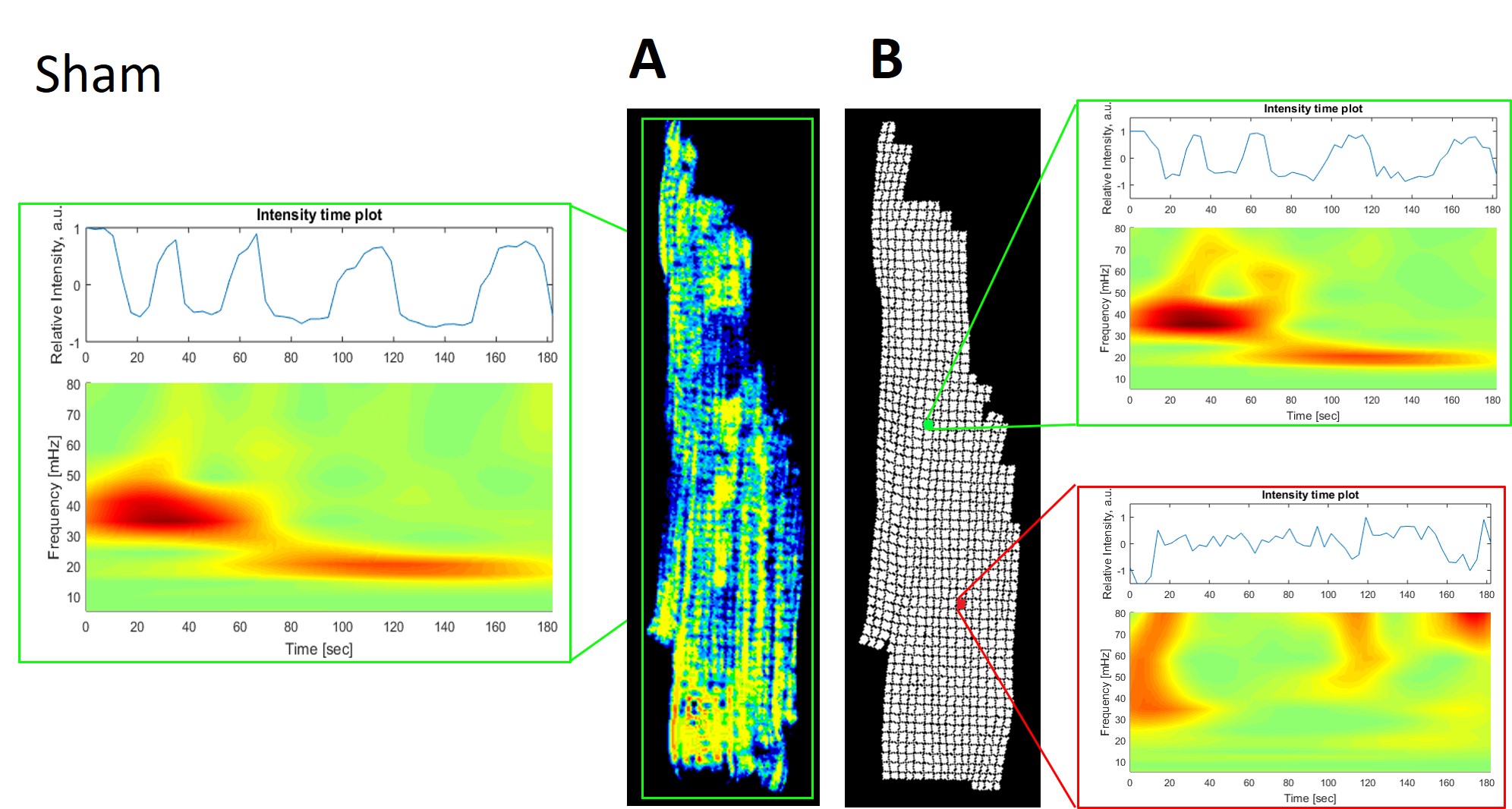

Supplement: FIGURE S3 — Wavelet analysis in a diabetic ventricular myocyte (STZ). Depicted is the mitochondrial network of a STZ cell (center left), and its respective grid with each element corresponding to individual mitochondria (center right). The absolute squared wavelet transform over frequency and time of the TMRM signal of the whole cardiomyocyte (A, left panel) or individual mitochondria (B) belonging to the spanning cluster (B, top panel) or not (B, bottom panel), are represented. There are higher frequencies up to ∼80 mHz of the spanning cluster when compared with the wavelet analysis of the control (sham) cell (Supplementary Figure S2). This is in agreement with the analysis in Figure 2. [file Image_3.jpeg]

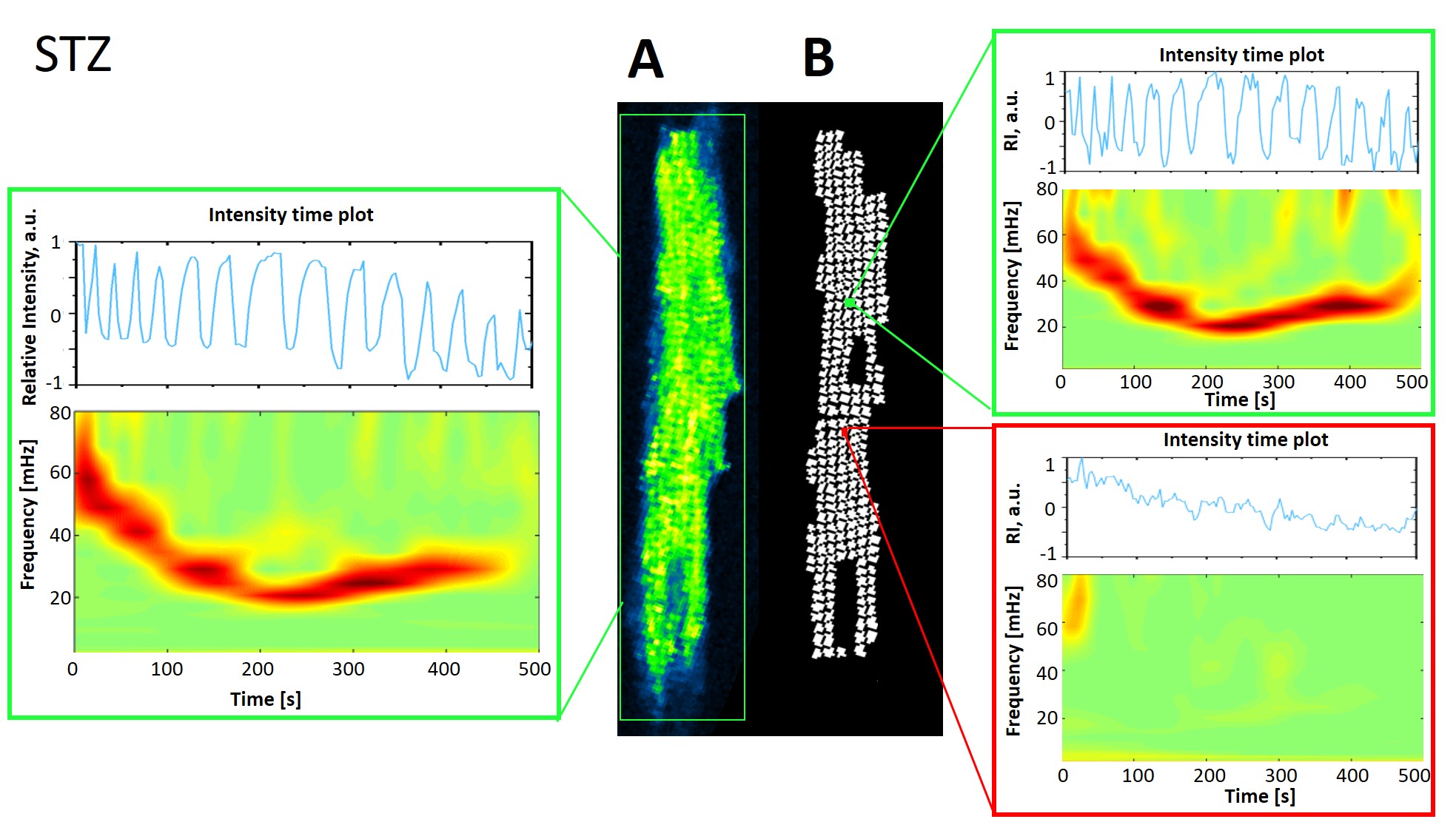

Supplement: FIGURE S4 — Wavelet analysis in a diabetic ventricular myocyte after insulin treatment (STZ + Ins). Mitochondrial network of a STZ + Ins cell (center left), and its respective grid with each element corresponding to individual mitochondria (center right). The absolute squared wavelet transform over frequency and time of the TMRM signal of the whole cardiomyocyte (A, left panel) or individual mitochondria (B) belonging to the spanning cluster (B, top panel) or not (B, bottom panel), are represented. There are mostly lower frequencies ∼20 mHz of the spanning cluster when compared with the wavelet analysis of the STZ cell (Supplementary Figure S3). This is in agreement with the analysis in Figure 2. [file Image_4.jpeg]
